# The Ontogeny of Anxiety-Like Behavior in Rats from Adolescence to Adulthood

**DOI:** 10.1002/dev.20468

**Published:** 2010-05-12

**Authors:** Debra A Lynn, Gillian R Brown

**Affiliations:** School of Psychology University of St Andrews South StreetSt Andrews Fife KY16 9JP, UK

**Keywords:** adolescence, anxiety, rats, open field, elevated plus-maze, sex differences

## Abstract

In human beings, susceptibility to anxiety disorders can be relatively high during adolescence. Understanding the ontogeny of anxiety-like behavior in laboratory rodents has implications for developing anxiolytic drugs that are suitable for this age group. Given the dearth of information about adolescent rodents, this study examined the response of both male and female adolescent, late adolescent, young adult, and older adult rats to three tests of anxiety-like behavior: the emergence test (ET), open field (OF), and elevated plus-maze (EPM). The results showed that adolescent rats exhibited a higher anxiety-like response than adults on each test; the amount of locomotion in the OF and percentage of time spent on the open arms of the EPM increased across the age groups, while older adult rats made the fewest start box re-entries in the ET. These results support the hypothesis that adolescent rats have a more pronounced response to stressors than do adults. © 2010 Wiley Periodicals, Inc. Dev Psychobiol 52: 731–739, 2010.

## INTRODUCTION

Adolescence is a period of heightened susceptibility to anxiety disorders for some individuals (Crick & Zahn-Waxler, 2003; Paus, Keshavan, & Giedd, 2008; Petersen, 1988), potentially due to the developmental changes in gonadal and adrenal hormone production that accompany this stage of life (Hayward & Sanborn, 2002; McCormick & Mathews, 2007; Reardon, Leen-Feldner, & Hayward, 2009; Spear, 2000a,b). The development of new anxiolytic drugs for adolescents is important given that currently available anxiolytic drug treatments can have significant negative side effects in this age group (Hammad, Laughren, & Racoosin, 2006; Malkesman et al., 2009). While research on anxiety-like behavior in laboratory animals has important implications for the development of new drugs, relatively little is known about the development of anxiety-like behavior in rodents. The aim of this study was, therefore, to investigate the ontogeny of anxiety-like behavior in rats from adolescence to adulthood.

Anxiety-like behavior in rodents has commonly been assessed by recording how animals respond to a novel, potentially threatening environment (Litvin, Pentkowski, Pobbe, Blanchard, & Blanchard, 2008). In this study, we measured the response of rats to three novel pieces of apparatus: the emergence test (ET), the open field (OF) and the elevated plus-maze (EPM). All of these tests assume that rats find novel, exposed environments relatively aversive due to potential predation risk. The ET consists of a box or canopy that is placed into a novel arena (Grewel, Shepherd, Bill, Fletcher, & Dourish, 1997), and a fast emergence from the canopy is described as a low anxiety-like response (Ray & Hansen, 2004). The OF consists of a novel, enclosed space into which the animal is placed for a fixed amount of time (Hall, 1934, 1936), and high levels of locomotion in the center of the OF is described as a low anxiety-like response (Prut & Belzung, 2003). The EPM consists of two open and two enclosed arms arranged in a plus-shape and raised above the ground (Handley & Mithani, 1984; Montgomery, 1955), and individuals that spend most time on the open arms of the EPM are assumed to exhibit the lowest anxiety-like response (Walf & Frye, 2007). Treatment of rodents with anxiolytic agents has been used to validate these behavioral tests, with treated animals emerging from a box or canopy sooner, and spending more time in the center of the OF and on the open arms of the EPM, than control animals (Hogg, 1996; Prut & Belzung, 2003; Yang, Gorman, & Dunn, 1999).

Relatively few studies have compared the behavior of adolescent and adult rodents on these traditional behavioral tests of anxiety-like responses, and such studies have produced conflicting results. In the OF, adolescent rodents have been reported to exhibit either higher levels (e.g., Arakawa, 2005; Bronstein, 1972; Philpot & Wecker, 2008; Stansfield & Kirstein, 2006) or lower levels (e.g., Candland & Campbell, 1962) of locomotor exploration than adults, and, in the EPM, adolescents have been reported to spend either more time (e.g., Doremus-Fitzwater, Varlinskaya, & Spear, 2009a; Macri, Adriani, Chiarotti, & Laviola, 2002) or less time (e.g., Doremus, Brunell, Varlinskaya, & Spear, 2003) on the open arms than adults. Other studies have failed to find age differences in performance on these pieces of apparatus (e.g., Arakawa, 2005; Hefner & Holmes, 2007). In general, age differences in anxiety-like behavior have not been the main focus of these studies, methodological differences between studies are likely to have contributed to the conflicting results, and most studies have used only one behavioral test (Doremus, Varlinskaya, & Spear, 2004; Doremus-Fitzwater et al., 2009a; Slawecki, 2005). Measuring the behavioral response of adolescent and adult rats to multiple tests provides an opportunity to investigate in detail the relationship between age and anxiety-like behavior, and to examine whether these tests elicit similar or different anxiety-like responses from each other.

In this study, we examined whether anxiety-like behavior in rats increases, declines, or remains stable during the transition from adolescence to adulthood, as measured in the ET, OF, and EPM. We also tested whether male and female rats behave differently on these behavioral tasks. Adult female rodents have been reported to locomote more on the OF, spend more time on the open arms of the EPM, and emerge sooner in the ET than adult males (e.g., Johnston & File, 1991; Roman & Arborelius, 2009; Swanson, 1966). Sex differences in performance on these tasks could relate to sex differences in exploratory motivation or in spatial learning ability (Lynn & Brown, 2009). However, only a small number of studies have examined sex differences on these tests during adolescence. Some studies reported that adolescent female rodents locomote more than same-aged males in an OF (e.g., Beatty & Fessler, 1976; Stewart, Skvarenina, & Pottier, 1975), while others failed to find a sex difference in locomotion at this age (e.g., Masur, Schutz, & Boerngen, 1980; Slob, Huizer, & van der Werff ten Boesch, 1986). In the EPM, adolescent female rats have been reported to exhibit more open arm activity than males of the same age (e.g., Elliott, Faraday, Phillips, & Grunberg, 2004; Leussis & Anderson, 2008; Lynn & Brown, 2009), although other studies reported that this sex difference does not emerge until early adulthood (Estanislau & Morato, 2006; Imhof, Coelho, Schmitt, Morato, & Carobrez, 1993).

The studies discussed so far have generally only focused on two age points, thereby precluding a detailed analysis of the ontogeny of behavioral responses to tests of anxiety-like behavior. In our study, the behavioral responses of male and female rats were examined at four ages: adolescence (postnatal days (*pnd*) 34–39), late adolescence (pnd 51–55), young adulthood (pnd 65–69), and older adulthood (pnd 104–109). The adolescent period in rats has been defined as starting around pnd 28 (Spear, 2000a), coinciding with rising circulating gonadal hormone levels in both sexes (Gabriel, Roncancio, & Ruiz, 1992). Around pnd 35–40, females exhibit vaginal opening and irregular ovarian cycling, while testosterone levels rise gradually in males (Gabriel et al., 1992). During late adolescence (pnd 46–59; Tirelli, Laviola, & Adriani, 2003), females exhibit regular cycles, and males are capable of producing fertile sperm (Gabriel et al., 1992; Tentler et al., 1997). By pnd 60, rats are usually considered to be fully sexually mature. However, the male testes continue to develop into young adulthood (Knorr, Vanha-Perttula, & Lipsett, 1970), and testosterone levels peak around pnd 70 before falling to adult levels (Zanato, Martins, Anselmo-Franci, Petenusci, & Lamano-Carvalho, 1994). The four age points chosen for our study therefore represent the peak of adolescence through to full sexual maturity for both sexes. In combination with our previous research on anxiety-like behavior from early-to-late adolescence (Lynn & Brown, 2009), the results will provide detailed information about the ontogeny of anxiety-like behavior in rats from adolescence to adulthood.

During the adolescent period, the hypothalamic–pituitary–adrenal (HPA) axis undergoes maturational changes in rodents. For instance, juvenile rats exhibit a hyperresponsiveness of the HPA axis and a prolonged production of corticosterone following stress compared to adults (Romeo, 2005), and adolescence is likely to be a period during which the developing stress axis is highly sensitive to perturbations (Andersen, 2003). Given that developmental changes in both gonadal and adrenal hormone production have been hypothesizd to underlie the increased susceptibility of adolescents to anxiety disorders in human beings (Sanborn & Hayward, 2003), a fuller understanding of the ontogeny of anxiety-like behavior in male and female rodents will provide the basis for future investigations of the link between hormones and anxiety.

## METHODS

### Subjects and Housing

The subjects were 72 (35 males and 37 females) Lister-hooded rats (*Rattus norvegicus*) selected from nine litters bred in-house (breeding stock acquired from Harlan, Blackthorn, UK). The breeders and offspring were housed in a holding room (lights on 07:00–19:00; temperature: 20 ± 1°C; relative humidity: 55 ± 5%) in opaque plastic and wire mesh home cages (52 cm × 40 cm × 26 cm). Water and soy-free pelleted food were available ad libitum. The subjects were weaned into same-sex groups at pnd 21 and housed in same-sex sibling pairs or triplets at pnd 29.

Different groups of animals were used in each age category, as prior exposure to novel apparatus has been shown to influence later performance (e.g., Bertoglio & Carobrez, 2000). The following numbers of subjects were allocated to each age category: adolescence (pnd 34–39) = 9 males and 10 females; late adolescence (pnd 51–55) = 9 males and 8 females; young adulthood (pnd 65–69) = 9 males and 10 females; and older adulthood (pnd 104–109) = 8 males and 9 females. No more than two subjects of each sex were taken from a single litter within each age group. Each animal was handled only for the purpose of transporting between the holding room and testing room, and for weekly weighing and cage cleaning.

### Apparatus

The ET consisted of an area of vinyl floor (measuring 120 cm × 120 cm) enclosed on all four sides by a wooden, painted wall (measuring 50 cm in height). A round, opaque plastic box (18 cm × 14.3 cm) was placed mid-way along a wall of the arena with a single entrance/exit hole (8 cm × 7 cm) directed toward the center of the arena. At the beginning of the test, the subject was placed inside the box via a lid.

The OF was identical to the enclosure described above, minus the box. The floor of the arena was marked into nine areas (eight outer and one central area) by four lines, each 30 cm from one of the walls. At the beginning of each test, the subject was placed into the front left corner of the arena.

The EPM consisted of four painted, wooden arms (51 cm × 11 cm) raised 56 cm from the ground on a metal frame. Two of the arms had gray wooden walls (closed arms; 40 cm high) and the remaining two arms lacked walls (open arms), and the central area was 11 cm^2^. At the start of the test, the subject was placed into the central area facing a closed arm.

### Experimental Design

The subjects completed the three tests in the following order: ET, OF, and EPM. All subjects received the tests in the same order, so that any possible order effects were uniformly distributed across age groups and variance between individuals on each task was minimized. The animals completed one test per day for three consecutive days, and all tests were conducted between 11:00 and 15:00 hr in the same testing room under dim, white light (approximately 25 lux). The apparatus was surrounded by a black curtain to reduce the number of external cues visible to the subject, and a video camera attached to the ceiling relayed images to a computer. Immediately prior to a test, the subject was transported to the test room in a small, covered box. After each test, the subject was returned directly to the home-cage, and the apparatus was cleaned with a 70% alcohol solution.

Each test lasted 10 min, during which the following behavioral data were collected either by Ethovision XT software (Noldus Information Technology, Wageningen, The Netherlands) or by manual entry onto a laptop computer running in-house software. In the ET, the latency to exit the start box, the total number of times that the subject re-entered the box, and total peeks without exiting the box were recorded manually into the computer by the observer. A peek was defined as a subject putting its head out of the start box, up to the level of the ears, without exiting. In the OF, the number of entries into a new area was recorded manually, and an entry was defined as all four paws crossing a line. Total locomotion was calculated as the total number of line crossings; however, as line crossings have been suggested to be an unsuitable method for comparing locomotor activity across ages (Vila, Philpot, & Kirstein, 2004), Ethovision was also used to calculate total distance moved (TDM; cm) and percentage of time spent mobile. Rears were also manually recorded into the computer, with a rear being defined as an animal sitting on its hindlegs and stretching itself upwards. In the EPM, the percentage of time spent on the open arms and the percentage of open arm entries (relative to total line crossings) were recorded manually, in addition to rears and peeks onto the open arms (as defined in the ET and OF). Ethovision also provided information on the TDM and the percentage of time spent mobile in the EPM.

### Statistical Analyses

The behavioral data were checked for normality using the Kolmogorov–Smirnov test. Data not normally distributed were subject to a log transformation. Normal and normalized data were analyzed using multivariate analyses of variance (MANOVA) with age and sex as between-subject variables and the above behavioral measures as within-subject variables. Variables not normally distributed were analyzed using Kruskal–Wallis tests. Planned post hoc trend tests (polynomial linear/quadratic contrasts) were performed to examine patterns of behavioral change from adolescence to adulthood. Where there was a main effect of age on locomotion measures (TDM and total line crossings in the OF), an analysis of covariance (ANCOVA) was performed using body weight as a covariate. Age × sex interactions are only reported if significant. Pearson's correlation coefficient tests were used to examine the relationship between measures on the behavioral tests. Body weight data were analyzed using a univariate ANOVA with age and sex as between-subjects variables. The data reported in parentheses are means ± standard errors (SEMs). SPSS Version 17.0 was used for data analysis, and an alpha level of <.05 was used throughout.

## RESULTS

### Weight Data

The analyses revealed significant main effects of age (*F*_3,64_ = 43.91, *p* < .001) and sex (*F*_1,64_ = 44.32, *p* < .001), with weight increasing with age and males weighing more than females (Tab. 1). Age and sex also significantly interacted (*F*_3,64_ = 9.00, *p* < .001), as male rats gained more weight than did females.

**Table 1:** The Mean Weight (g) of Male and Female Rats in Each Age Group (Means ± SEMs)

	Male	Female
Adolescent (pnd 34–39)	109.0 ± 15.1	110.1 ± 14.3
Late adolescent (pnd 51–55)	200.7 ± 15.1	157.4 ± 16.0
Young adult (pnd 65–69)	266.9 ± 15.1	172.1 ± 14.3
Older adult (pnd 104–109)	351.0 ± 16.0	203.7 ± 15.1

### Emergence Test

- *Latency to exit the box*: There was a significant main effect of sex (*F*_1,22_ = 5.66, *p* = .026) on latency to exit, with females being quicker to leave the box than males (males: 13.6 ± 2.3 s; females: 10.0 ± 2.2 s). There was no main effect of age (*F*_3,22_ = 1.05, *p* = .392).
- *Total number of re-entries*: There was a significant effect of age on the total re-entries into the box (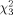 = 7.85, *p* = .049), but no effects of sex (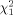 = .10, *p* = .919). Trend tests revealed a significant linear decrease in cage re-entries with age (*p* = .007; adolescents = 4.3 ± .4, late adolescents = 4.7 ± .4, young adults = 4.6 ± .4, older adults = 3.2 ± .4).
- *Total peeks from the box*: There was neither a main effect of age (*F*_3,22_ = 2.40, *p* = .096) nor of sex on total number of peeks (*F*_1,22_ = 1.22, *p* = .282).

### Open Field

**FIGURE 1:**
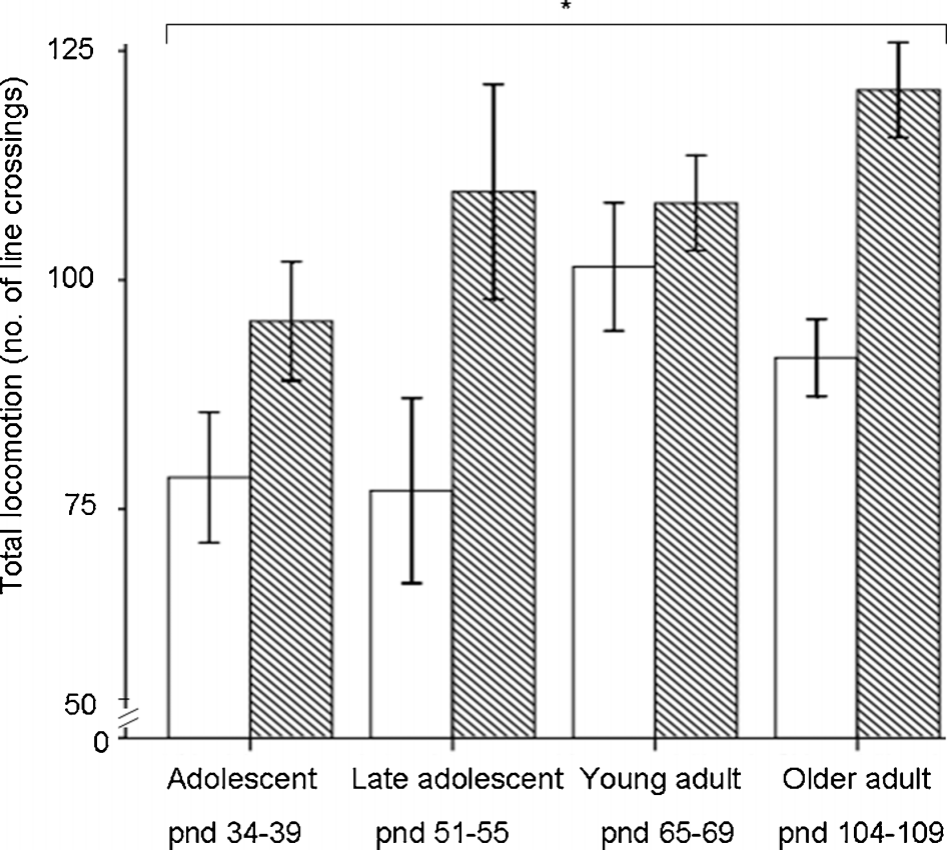
Total locomotion in the open field (means ± SEMs). Asterisk (*) indicates a significant main effect of age (*p* ≤ .05). White bars represent males; hatched bars represent females.

**FIGURE 2:**
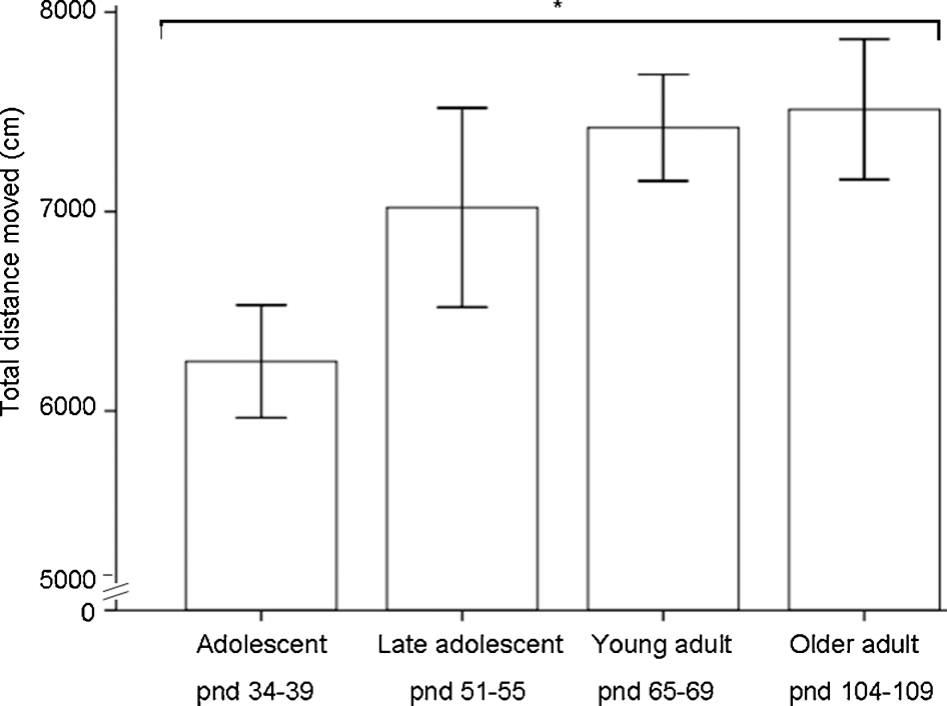
Total distance moved (cm) in the open field collapsed across subjects' sex (means ± SEMs). Asterisk (*) indicates a significant main effect of age (*p* < .05).

- *Total locomotion*: There was a significant main effect of sex (*F*_1,23_ = 8.10, *p* = .009), with females locomoting more than males, and a main effect of age (*F*_3,23_ = 3.51, *p* = .031). Trend analyses revealed a significant linear increase in total locomotion with age (*p* = .011; Fig. 1). After covarying body weight, the main effect of age on total locomotion persisted (*F*_3,66_ = 4.52, *p* = .006).
- *Total distance moved*: This measure did not differ between the sexes (*F*_1,23_ = 2.79, *p* = .108), but there was a main effect of age (*F*_3,23_ = 3.39, *p* = .035; Fig. 2). Trend analyses revealed that the TDM increased linearly with age (*p* = .011). This main effect of age on TDM persisted after considering body weight as a covariate (*F*_3,66_ = 3.04, *p* = .035).
- *Percentage of time spent mobile*: There was no main effect of age (*F*_3,23_ = .23, *p* = .874) or sex (*F*_1,23_ = 2.94, *p* = .100) on this measure.
- *Percentage of time in the center*: There was no main effect of age (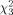 = 6.44, *p* = .092) or of sex (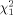 = .75, *p* = .385) on the percentage of time spent in the center of the OF.

### Elevated Plus-Maze

**FIGURE 3:**
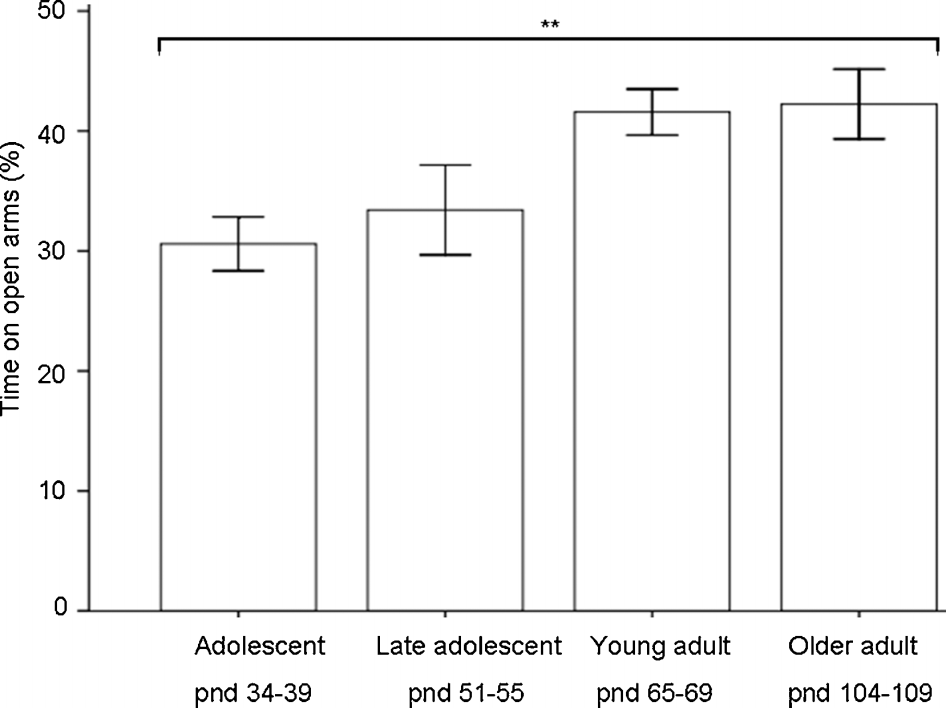
Percentage of time spent on open arms collapsed across subjects' sex (means ± SEMs). **A significant main effect of age (*p* < .01).

- *Percentage of time on open arms*: There was a significant main effect of age on the percentage of time on the open arms (*F*_3,23_ = 6.56, *p* = .002; Fig. 3). Trend analyses revealed that the percentage of open arm time increased linearly with age (*p* = .001). There was no main effect of sex (*F*_1,23_ = 2.25, *p* = .147).
- *Percentage of open arm entries*: There was no main effect of sex (*F*_1,23_ = 1.40, *p* = .088), but there was a significant main effect of age (*F*_3,23_ = 3.09, *p* = .047), with the percentage of open arm entries linearly increasing with age (*p* = .007; adolescents = 21.6 ± 1.2, late adolescents = 21.6 ± 1.3, young adults = 24.4 ± 1.2, older adults = 25.7 ± 1.3).

### Correlations Between Performance on the ET, OF, and EPM

The percentage of time on the open arms of the EPM was positively correlated with all locomotory measures on the OF: total locomotion (*ρ* = .292, *p* = .016), TDM (*ρ* = .350, *p* = .003), and the percentage of time spent mobile (*ρ* = .307, *p* = .011). The percentage of entries onto the open arms also tended to positively correlate with total locomotion (*ρ* = .221, *p* = .069) and TDM (*ρ* = .222, *p* = .069) on the OF. The percentage of time spent on the open arms of the EPM negatively correlated with the number of box re-entries on the ET (*ρ* = −.271, *p* = .026). The percentage of entries onto the open arms of the EPM also negatively correlated with ET box re-entries (*ρ* = −.266, *p* = .028) and tended to correlate negatively with latency to exit the ET box (*ρ* = −.232, *p* = .057).

## DISCUSSION

The aim of this study was to investigate the ontogeny of anxiety-like behavior in rats from adolescence to adulthood. The results indicated that the amount of exploratory behavior and measures of anxiety-like behavior decreased across the age groups on all three behavioral tests. More specifically, the amount of locomotion in the OF, as measured by line crossings or distance moved, increased from adolescence to adulthood; the amount of time on the open arms and number of open arm entries in the EPM increased from adolescence to adulthood; and older adults made the fewest re-entries into the start box in the ET. Our results are in line with previous reports of lower levels of exploration by adolescents in the OF (Candland & Campbell, 1962) and lower levels of open arm activity in the EPM compared to adults (Doremus et al., 2003). While animals at the beginning of adolescence (pnd 28) were not tested here, it is considered that the linear increases in exploration and anxiety-like behavior from adolescence to adulthood would hold, given previous research demonstrating lower locomotory and anxiety-like behavior in animals during early adolescence compared to their older adolescent counterparts (Hefner & Holmes, 2007; Lynn & Brown, 2009). In contrast, the results do not support the suggestion that adolescent rodents are more exploratory and less anxious than adults (e.g., Macri et al., 2002; Philpot & Wecker, 2008; Stansfield & Kirstein, 2006), or the general conclusion that adolescent rodents are more risk-taking and responsive to novelty than adults (Spear, 2007). Here, we examine possible explanations for the differences between studies.

One potential methodological difference between studies is whether the apparatus has been scaled for body size. Previous researchers have suggested that when comparing the behavior of adolescent and adult rodents, the apparatus should be scaled to take account of the size differences between age groups (Philpot & Wecker, 2008). Philpot and Wecker (2008) reported that adolescent male rats (pnd 31) spent a similar amount of time locomoting as late adolescents (pnd 56) in a scaled OF apparatus but reported that adolescents moved a greater total distance than the older animals. While our results also indicated that the amount of time spent locomoting did not differ with age, adolescents in our study moved a lesser total distance compared to adults, whether measured by line crossings or by tracking TDM. These age differences were maintained when body weight was included as a covariate in the analyses. The opposite result of Philpot and Wecker (2008) suggests that adolescent novelty induced locomotion is enhanced when the apparatus is scaled to size. However, it is still unclear whether scaling relative to crown-rump length results in two pieces of apparatus that are equivalent from the perspective of the subjects. The use of multiple behavioral tests, where appropriate, can avoid over-reliance on a single piece of apparatus that can vary greatly in design (Crabbe & Morris, 2004).

Consistent with our OF data, the amount of time spent on the open arms of the EPM increased across age groups. A previous study has presented a factor analysis of adolescent and adult EPM performance, which suggested that similar components underlie the behavioral response of these two age groups (Doremus, Varlinskaya, & Spear, 2006). In line with our results, Doremus et al. (2003) reported that adolescent rats spend less time on the open arms than adults using apparatus that was scaled to the relative sizes of the subjects. Therefore, the age difference in EPM performance appears robust to changes in relative apparatus size. Our results also indicate that anxiety-like responses are similar in all three pieces of apparatus; for example, time spent on the open arms of the EPM correlated positively with OF locomotor activity and negatively with the number of box re-entries in the ET. The effects of age were similar across all three tests, which strengthens our conclusion that anxiety-like behavior declines from adolescence to adulthood in rats. The lack of age changes in the percentage of time in the center of the OF, and latency to exit the start box in the ET, are likely to be due to floor effects in the data, with all animals exhibiting very low numbers of entries into the center of the OF and short latencies to exit the ET start box.

In this study, we compared the performance of males and females across the age groups. Our results indicated that females are quicker to leave the start box than males in the ET and locomote more than males in the OF when measured by line crossings. These findings are in line with sex differences in adult performance on these tests (e.g., Johnston & File, 1991; Roman & Arborelius, 2009; Swanson, 1966), and the OF results support previous studies of adolescents (e.g., Beatty & Fessler, 1976; Lynn & Brown, 2009; Stewart et al., 1975). The lack of age × sex interactions in our data sets precludes us from drawing any conclusions about the age of emergence of sex differences in performance on these tasks. Previous researchers have concluded that male rodents are more anxious, or more fearful, than females based on response to novel apparatus such as the OF and EPM (e.g., Aguilar et al., 2003; Zimmerberg & Farley, 1993). However, alternative explanations for the behavioral sex differences also exist, including that females exhibit a higher motivation than males to investigate and/or escape from novel environments (Brown & Nemes, 2008). Distinguishing between these alternative explanations will be difficult based on behavioral measures alone (Hughes, 1997).

In contrast to a previous study in our laboratory (Lynn & Brown, 2009), no sex differences in EPM performance were found in the current study. The previous study compared the performance of male and female adolescent rats on both the OF and EPM with similar numbers of subjects as in the current study. However, a key difference could have been the time between tests. In the earlier study (Lynn & Brown, 2009), each test was conducted two to three days apart, whereas the current study exposed the subjects to the three tests over three consecutive days. Previous research has indicated that EPM performance is particularly sensitive to prior testing in other apparatus (Carobrez & Bertoglio, 2005) and that pretest manipulation reduces the extent of the sex differences in EPM performance (Doremus-Fitzwater et al., 2009a). Further experiments are currently underway in our laboratory to examine the effects of repeated behavioral testing on the response of adolescent and adult rats to novel environments.

The main conclusion to be drawn from this study is that anxiety-like behavior on traditional behavioral tests gradually decreases from adolescence into adulthood in rats. Therefore, adolescent rodents could provide relevant information about the mechanisms by which susceptibility to anxiety disorders changes from adolescence to adulthood in human beings. The gradual changes in performance of rats on the ET, OF, and EPM correspond to changes in adrenal hormone production over these ages. For instance, the HPA axis undergoes developmental changes from hyperresponsiveness in the prepubertal animals to lowered responsiveness in adulthood (Romeo, 2005). A growing number of studies have shown that exposure to stress has a more pronounced and long-lasting effect on adolescent than adult rodents (e.g., Doremus-Fitzwater, Varlinskaya, & Spear, 2009b; McCormick, Smith, & Mathews, 2008; Stone & Quartermain, 1997; for reviews, see McCormick & Mathews, 2007; Romeo, 2010). In addition, human adolescence is associated with heightened basal and stress-induced activity of the HPA axis (Lupien, McEwen, Gunnar, & Heim, 2009). Therefore, adolescence is likely to be a period during which the developing stress axis is potentially highly sensitive to perturbations, and exposure to stress during this period of life could potentially have life-long consequences for mental health in human beings.

## NOTES

This research was funded by the Wellcome Trust (Research Career Development Fellowship awarded to G.B.) and the School of Psychology, University of St Andrews. We thank the technical staff in the School of Psychology for support, Kevin Laland and two anonymous reviewers for comments on the manuscript.
